# Crowned Dens Syndrome Mimicking Septic Arthritis in a Renal Transplant Recipient

**DOI:** 10.7759/cureus.116735

**Published:** 2026-09-22

**Authors:** Harishankar Praveen Nair, Hassan Butt, Marcus Au, Jonathan Heaney

**Affiliations:** 1 Rheumatology, Newcastle upon Tyne Hospitals NHS Foundation Trust, Newcastle upon Tyne, GBR

**Keywords:** calcium pyrophosphate deposition disease (cppd), chondrocalcinosis, crowned dens syndrome, crystal arthropathy, inflammatory neck pain, renal transplantation, tacrolimus

## Abstract

Crowned dens syndrome (CDS) is a rare manifestation of calcium pyrophosphate deposition (CPPD) disease, characterized by crystal deposition surrounding the odontoid process. It typically presents with acute neck pain, restricted cervical movement, and elevated inflammatory markers, frequently mimicking infection or other inflammatory conditions.

We report a 66-year-old man with end-stage renal disease secondary to congenital obstructive uropathy and a cadaveric renal transplant, maintained on tacrolimus and prednisolone. He presented with worsening left wrist pain and swelling accompanied by severe neck pain and marked restriction of cervical movement. Elevated inflammatory markers and peripheral joint inflammation initially raised concern for septic arthritis, prompting joint aspiration and empirical intravenous antibiotics. Persistent neck pain led to computed tomography (CT) of the cervical spine, which demonstrated characteristic peri-odontoid calcification consistent with CDS. Magnetic resonance imaging (MRI) demonstrated mild thickening of the cruciate ligaments and excluded cervical cord compression. Radiographs of the left hand and wrist demonstrated soft-tissue calcification consistent with peripheral CPPD. Following multidisciplinary rheumatology and nephrology input, he was treated with colchicine and an increased corticosteroid dose, resulting in rapid clinical improvement and near-complete resolution of symptoms.

This case demonstrates an important diagnostic challenge in renal transplant recipients. Chronic immunosuppression increases susceptibility to infection, making prompt exclusion of septic arthritis essential in patients presenting with an acutely swollen joint. However, chronic kidney disease and associated abnormalities of mineral metabolism also predispose to CPPD. CDS should therefore remain an important differential diagnosis when significant neck pain and restriction persist despite initial treatment. CT is particularly valuable for demonstrating characteristic peri-odontoid calcification, while MRI provides complementary assessment when neurological or alternative spinal pathology is suspected.

Renal transplantation should not narrow the differential diagnosis to infection alone. CDS should be considered in patients with persistent acute neck pain and risk factors for CPPD once serious infection has been appropriately investigated. Early CT imaging and multidisciplinary management can facilitate diagnosis, enable appropriately tailored anti-inflammatory therapy, and avoid unnecessary prolonged antimicrobial treatment or invasive investigation.

## Introduction

Acute neck pain associated with fever and elevated inflammatory markers presents a significant diagnostic challenge in acute medical practice. Although infectious, inflammatory, vascular, and neurological disorders are commonly considered, crystal-induced arthropathies involving the atlantoaxial articulation remain an under-recognized cause of this clinical presentation [1-3]. Crowned dens syndrome (CDS), first described by Bouvet et al. [2] in 1985, represents a rare manifestation of calcium pyrophosphate deposition (CPPD) disease, characterized by calcification of the transverse, alar, and cruciform ligaments surrounding the odontoid process, producing the characteristic “crown-like” appearance on computed tomography (CT) imaging [1,3].

CPPD is a crystal arthropathy characterized by the deposition of calcium pyrophosphate crystals, most commonly affecting fibrocartilage and hyaline cartilage of large joints such as the knees, wrists, hips, and shoulders [1,4,5]. The true prevalence of CDS remains uncertain because many patients are initially investigated using magnetic resonance imaging (MRI), which is relatively insensitive for detecting peri-odontoid calcification [3,6,7]. Consequently, the syndrome is likely underdiagnosed, particularly among older adults presenting with non-specific neck pain [3,8].

Clinically, CDS typically presents with abrupt onset of severe neck pain, marked restriction of cervical movement, fever, and elevated inflammatory markers. These features frequently mimic bacterial meningitis, cervical spondylodiscitis, epidural abscess, polymyalgia rheumatica, giant cell arteritis, metastatic disease, or septic arthritis, often resulting in extensive investigation and empirical antimicrobial treatment before the diagnosis is established [2,8]. Recognition is important because symptoms usually respond rapidly to anti-inflammatory therapy, thereby avoiding unnecessary invasive investigations and prolonged antibiotic exposure [1,4,8]. Renal transplantation represents a particularly challenging clinical setting, as immunosuppression appropriately lowers the threshold for suspecting infection, while chronic kidney disease (CKD)-associated disturbances in mineral metabolism, including secondary hyperparathyroidism and abnormalities in magnesium homeostasis, may promote CPPD crystal formation and deposition [1]. Reports of CDS occurring in renal transplant recipients remain limited, and the interaction between immunosuppression, altered calcium-phosphate homeostasis, and crystal arthropathy has not been well-characterized [8].

We present a case of CDS in a renal transplant recipient receiving tacrolimus whose initial presentation strongly suggested septic arthritis because of concomitant wrist involvement and a marked systemic inflammatory response. Through a focused review of the literature, we discuss the pathophysiology, diagnostic pitfalls, imaging characteristics, and current management strategies for this uncommon but clinically important condition.

## Case presentation

We report the case of a 66-year-old man who presented with worsening pain and swelling in his left wrist, together with neck pain and restricted cervical movement. His medical history included end-stage renal disease, cadaveric renal transplantation in 2006, maintained on tacrolimus, hypertension, and gout. His medication history included aspirin 75 mg, atorvastatin 80 mg, tacrolimus 4 mg, and prednisolone 5 mg.

Despite management of the wrist pathology, significant neck pain persisted without a clear explanation. Admission blood tests demonstrated a marked inflammatory response, with CRP elevated at 200 mg/L (reference range <5 mg/L) and a WBC count of 11.74 × 10⁹/L (reference range 4.0-11.0 × 10⁹/L). Serial inflammatory markers subsequently improved during treatment, with CRP decreasing to 2 mg/L (reference range <5 mg/L) at follow-up.

CT of the cervical spine demonstrated calcification within the cruciate ligament posterior to the odontoid peg, with overall appearances suggestive of calcium pyrophosphate deposition disease and CDS (Figure 1A). The patient also reported bilateral arm pain radiating from the neck, raising suspicion of cervical cord compression. MRI demonstrated mild thickening of the cruciate ligaments surrounding the odontoid process and excluded cervical cord compression (Figure 1B). In conjunction with the characteristic CT findings, these appearances supported the diagnosis of CDS.

**Figure 1:**
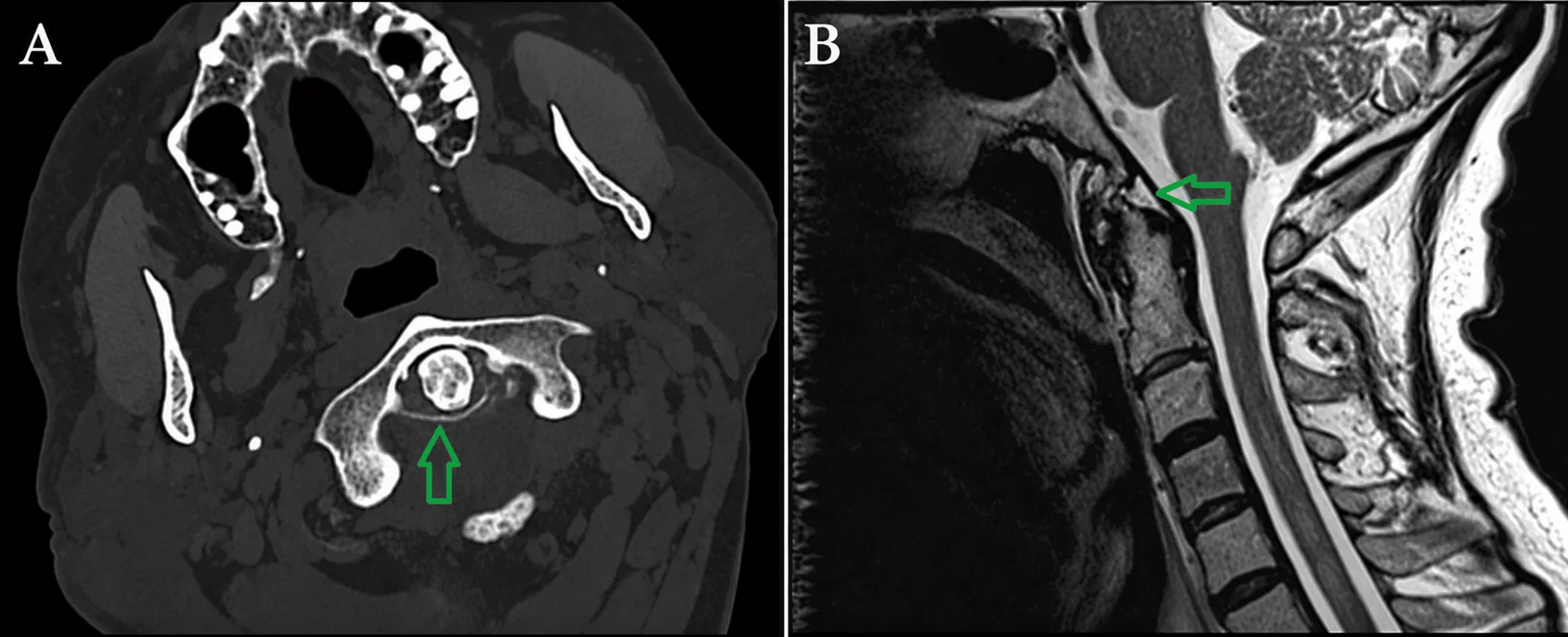
CT and MRI of the cervical spine demonstrating peri-odontoid calcification. (A) Coronal CT showed calcification within the cruciate ligament posterior to the odontoid peg (arrow), with appearances consistent with calcium pyrophosphate deposition disease and crowned dens syndrome. (B) Sagittal T2-weighted MRI showed thickening of the cruciate ligament posterior to the odontoid peg (arrow).

Radiographs of the left hand and wrist demonstrated faint soft-tissue calcification consistent with peripheral CPPD (Figure 2).

**Figure 2:**
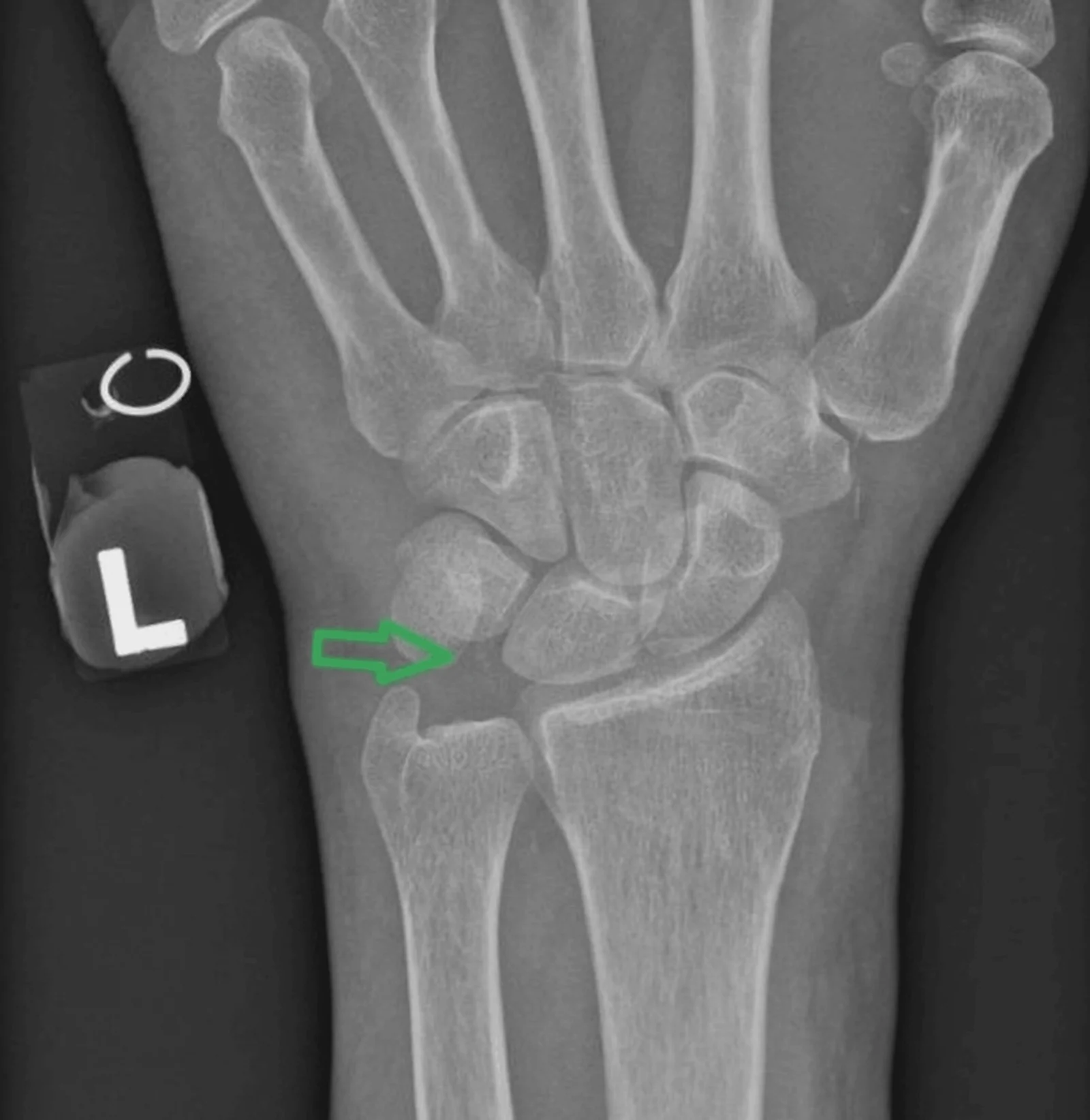
Radiograph of the left wrist. The arrow indicates faint soft-tissue calcification adjacent to the triquetrum, consistent with peripheral calcium pyrophosphate deposition disease. "L" denotes the standard radiographic laterality marker.

Following multidisciplinary discussion involving the rheumatology and nephrology teams, the patient was treated conservatively with colchicine 500 µg twice daily for three days and an increased corticosteroid dose. Serial inflammatory markers demonstrated progressive improvement, with near-complete resolution of symptoms following treatment. A summary of the investigations, imaging findings, and differential diagnoses considered is presented in Table 1.

**Table 1:** Summary of investigations, imaging, and differential diagnoses. CRP: C-reactive protein, WBC: white blood cell, CT: computed tomography, MRI: magnetic resonance imaging, CPPD: calcium pyrophosphate deposition disease.

Category	Investigation/finding	Interpretation
Blood tests	CRP 200 mg/L (admission; reference range <5 mg/L)	Marked acute-phase response
	WBC 11.74 ×10⁹/L (reference range 4.0-11.0 ×10⁹/L)	Mild leukocytosis
	CRP 2 mg/L (post-treatment; reference range <5 mg/L)	Near-complete biochemical resolution
	Renal function (stable, transplanted kidney)	No evidence of graft dysfunction or sepsis-related deterioration
Microbiology	Left wrist joint aspirate	No organisms isolated; culture-negative
Imaging	CT cervical spine (Figure 1A)	Calcification within the cruciate ligament posterior to the odontoid peg, consistent with CPPD and crowned dens syndrome
	MRI cervical spine (Figure 1B)	Mild thickening of the cruciate ligaments surrounding the odontoid process; no cervical cord or nerve root compression
	Plain radiograph, left hand and wrist (Figure 2)	Faint soft-tissue calcification consistent with peripheral CPPD
Differential diagnoses considered	Septic arthritis, meningitis, cervical discitis, epidural abscess, giant cell arteritis, rheumatoid arthritis, crystal arthropathy, crowned dens syndrome	Alternative diagnoses considered less likely based on culture-negative aspirate, absence of meningism, cross-sectional imaging, and clinical response to anti-inflammatory therapy

## Discussion

This case highlights the clinical challenge of managing renal transplant recipients. Long-term immunosuppression increases susceptibility to infection, making septic arthritis an important differential diagnosis in a patient presenting with an acutely swollen joint. Nevertheless, clinicians should recognize that renal transplant recipients may also have risk factors for CPPD, and failure to consider this diagnosis may delay appropriate treatment.

Multiple recognized risk factors for CPPD were present in our patient. He had a baseline eGFR of 26 mL/min/1.73 m² (reference range >90 mL/min/1.73 m²), together with persistently elevated parathyroid hormone levels over many years, representing potential metabolic risk factors for CPP crystal deposition [1]. Tacrolimus is also associated with renal magnesium wasting and hypomagnesemia in renal transplant recipients [9]. However, only intermittent reductions in serum magnesium were observed in our patient, making CKD-related mineral dysregulation a more plausible contributor.

Management of CDS is generally conservative and focuses on controlling the acute inflammatory response. Non-steroidal anti-inflammatory drugs (NSAIDs), colchicine, and corticosteroids are established treatment options, with therapy tailored according to patient comorbidities [1,4]. In our patient, NSAIDs were avoided because of impaired renal function and renal transplantation. He responded rapidly to a short course of colchicine together with an increased corticosteroid dose, with resolution of symptoms and normalization of inflammatory markers. The characteristic peri-odontoid calcification on CT represented the strongest diagnostic evidence for CDS in this case, while the rapid response to colchicine and corticosteroids provided supportive evidence. Early recognition of CDS can therefore facilitate appropriate anti-inflammatory treatment while avoiding unnecessary prolonged antimicrobial therapy and invasive investigations [1,4,8].

The key clinical message is that renal transplantation should not narrow the differential diagnosis to infection alone. In patients with CKD-related mineral bone disease who develop persistent neck pain and elevated inflammatory markers, particularly when peripheral CPPD is present, early cervical CT should be considered. CT is the preferred modality for identifying the characteristic peri-odontoid calcification of CDS, while MRI can provide complementary assessment when neurological or alternative spinal pathology is suspected [3,6,7]. Close collaboration between rheumatology and nephrology can facilitate timely diagnosis and appropriately tailored management.

## Conclusions

CDS is an important differential diagnosis in renal transplant recipients presenting with acute neck pain and elevated inflammatory markers. Although infection must be promptly excluded in immunosuppressed patients, underlying CKD and associated mineral abnormalities may also predispose to CPPD. Early recognition of characteristic CT findings can facilitate timely diagnosis and appropriate anti-inflammatory treatment while avoiding unnecessary prolonged antimicrobial therapy and invasive investigations. Multidisciplinary collaboration between rheumatology and nephrology is particularly important for tailoring treatment to renal function and comorbidities.
